# Pathophysiology and Management of Placenta Accreta Spectrum

**DOI:** 10.3390/jdb13040045

**Published:** 2025-12-10

**Authors:** Lana Shteynman, Genevieve Monanian, Gilberto Torres, Giancarlo Sabetta, Deborah M. Li, Zhaosheng Jin, Tiffany Angelo, Bahaa E. Daoud, Morgane Factor

**Affiliations:** 1 Renaissance School of Medicine, Stony Brook University, Stony Brook, NY 11794, USA; 2 Department of Anesthesiology, Stony Brook University, Stony Brook, NY 11794, USA

**Keywords:** placenta accreta spectrum, postpartum hemorrhage, maternal morbidity, decidualization, extravillous trophoblasts, extracellular matrix remodeling, epithelial-to-mesenchymal transition (EMT), preoperative planning

## Abstract

Placenta Accreta Spectrum (PAS) disorders, including placenta accreta, increta, and percreta, are serious obstetric conditions characterized by abnormal placental adherence to the uterine wall. With increasing incidence, PAS poses significant risks, primarily through massive hemorrhage during or after delivery, often necessitating hysterectomy. Key risk factors include prior cesarean sections, uterine surgery, and placenta previa diagnosis. In this review, we will examine the pathophysiology of PAS, with a focus on the mechanisms underlying abnormal trophoblast invasion and defective decidualization. We will highlight the role of uterine scarring, extracellular matrix remodeling, dysregulated signaling pathways, and immune and vascular alterations in disrupting the maternal-fetal interface, ultimately predisposing to morbid placentation and delivery complications. We will also discuss the life-threatening complications of PAS, such as shock and multi-organ failure, which require urgent multidisciplinary intensive care, as well as the optimization of management through preoperative planning and intraoperative blood loss control to reduce maternal morbidity and mortality.

## 1. Introduction

Placenta Accreta Spectrum (PAS) disorders are rare but serious obstetric conditions with significant implications for maternal and fetal health. The most critical and common complication associated with PAS is massive hemorrhage, which often requires surgical intervention, typically involving hysterectomy [1]. The rising incidence of PAS, coupled with the significant number of cases that remain undiagnosed until birth, necessitates a high index of suspicion for PAS, especially when faced with massive hemorrhage in at-risk patients.

PAS encompasses a spectrum of conditions characterized by abnormal adherence of the placenta to the uterine wall, where increasing depth of placental invasion is associated with increased morbidity and mortality [2]. The clinical classification of PAS, developed by the International Federation of Gynecology and Obstetrics (FIGO), was published in 2019 as a general classification to standardize criteria for PAS internationally. The criteria are as follows:

- Grade 1: Abnormally adherent placenta (placenta adherenta or creta): attached directly to the myometrium without invasion.
- Grade 2: Abnormal invasion of placenta into the myometrium (placenta increta).
- Grade 3: Abnormal invasion of placenta through the full thickness of the uterine wall to the serosa, or beyond to surrounding pelvic tissues, vessels, and organs (placenta percreta) [3].

Other PAS classification systems include the Placenta Accreta Index, developed by Rac et al., which scores PAS risk based on key predictors found on ultrasound: number of prior cesarean deliveries, placental location, lacunar spaces, myometrial thickness, and bridging vessels [4]. This index will be further discussed in the Diagnosis subsection. PAS can also be classified by its characteristic macroscopic and histologic features that correspond to each grade of abnormal placentation. Similarly to the FIGO system, the histologic subcategories in Table 1, proposed by an expert group of panelist representatives of multiple gynecologic societies, correlate with the degree of invasion and local tissue destruction [5].

### 1.1. Incidence

The incidence of PAS has increased over the past few decades, largely driven by the increase in cesarean delivery rates and improved diagnostic techniques [4]. Previous studies have demonstrated a rise in incidence from as low as 1 in 2562 to 4027 in the late 1970s to 1 in 731 by the late 2000s [5]. More recent data from a national retrospective observational cohort study (2015–2017) involving 2.7 million cesarean deliveries reported a PAS diagnosis in approximately 1 in 313 women undergoing cesarean delivery [6]. Conversely, a similar study of over 8.6 million vaginal deliveries from 2016 to 2019 found an incidence rate of 1 in 3797 deliveries where PAS was not previously suspected [7]. A comprehensive meta-analysis comprising 7001 cases of PAS across 5.7 million cesarean and vaginal deliveries found significant variation in the overall prevalence rate, with an overall pooled prevalence of 0.17% (95% CI, 0.14–0.19) [8].

PAS is also becoming more prevalent among high-risk groups such as patients with a history of cesarean delivery and older maternal age. The incidence of PAS in the general population and within these cohorts was estimated to rise approximately 2% every three months in a three-year period [6]. Cases of PAS that involve invasion beyond the serosa are less common, with the incidence of adherent versus invasive subtypes estimated at 0.5 (95% CI 0.3–0.36) and 0.3 (95% CI 0.2–0.4) per 1000 births, respectively [8].

### 1.2. Risk Factors

Several risk factors significantly increase the likelihood of developing PAS, with previous cesarean deliveries and placenta previa among the most common.

A meta-analysis of approximately 30 million patients identified a history of cesarean section as a significant risk factor for PAS (Odds Ratio (OR) 2.95, 95% CI 1.32–6.60) [9]. Studies have consistently shown a strong correlation between the number of cesarean deliveries and the likelihood of PAS, with adjusted odds ratios increasing with each additional cesarean delivery. A large multicenter study of 73,257 cesarean deliveries in 2013 found that patients with one, two, or three prior cesarean deliveries had adjusted odds ratios for PAS of 2.9, 4.6, and 12.6, respectively (all *p* < 0.001) [10]. A similar study by Silver et al. in 2006 of 30,132 cesarean deliveries found that the risk of PAS reached 2% in patients having a fourth cesarean delivery and exceeded 6% in patients with six or more previous cesarean deliveries [11].

A recent meta-analysis of 46 studies comprising 2219 cases of PAS in 1,085,693 deliveries identified several additional potential risk factors for PAS, including obesity (OR 1.37), maternal age > 35 (OR 3.1), multiparity (OR 2.49), IVF (OR 2.8), and other uterine surgeries (OR 4.42) [12]. Other outcome measures that did not exhibit significance included smoking, previous abortion, and previous curettage. Although these factors contribute to the overall risk, their independent predictive value varies, and further large-scale studies are needed to better understand their impact.

The interplay between placenta previa and prior cesarean delivery remains the strongest predictor of PAS. Overall, 30 to 65% of PAS cases occur in women with this combined profile [13], and the likelihood of abnormal placentation rises steeply with increasing numbers of previous cesareans. While placenta previa without previous uterine surgery confers an approximate 10% risk of PAS, and prior cesarean in the absence of previa carries a risk near 12%, the combination increases this risk to roughly 25% with one prior cesarean and approaches 50% after two and 67% with four [14]. Consequently, screening strategies should concentrate on women with both previa and uterine scarring, as they represent the subgroup at greatest risk [15]. 

Studies focused specifically on this high-risk population demonstrate that additional clinical factors may further modify PAS risk. Prior postpartum hemorrhage (PPH), which may reflect impaired decidualization, has been associated with higher PAS incidence even among women who already have both previa and a prior cesarean [15]. These findings suggest that obstetric history beyond cesarean number may help refine risk stratification within this already vulnerable group.

Assisted reproductive technology also influences PAS risk. IVF pregnancies are associated with a higher risk of placenta previa, a major contributor to PAS. Therefore, the elevated PAS risk seen in IVF is largely explained by this increased likelihood of placenta previa. However, among women who already have both placenta previa and a prior cesarean, IVF itself does not further increase PAS risk [15].

A growing body of evidence highlights the importance of prior uterine procedures, particularly those that disrupt the endometrial–myometrial interface. One study by You et al. found that operative hysteroscopy and uterine curettage were important risk factors for PAS. Operative hysteroscopy, including lysis of intrauterine adhesions, endometrial ablation, hysteroscopic myomectomy, and septum resection, substantially increased PAS risk, with risk of PAS rising 2.4- to 5.4-fold when two or more procedures were performed [16]. Repeated uterine curettage demonstrated a similar dose-dependent association, with markedly elevated risk after three or more procedures [16]. In contrast, less invasive intrauterine interventions such as endometrial biopsy without curettage, simple diagnostic hysteroscopy, and endometrial polypectomy were found to not increase risk [16]. These distinctions underscore the need for clinicians to consider both the type and frequency of uterine procedures when evaluating PAS risk, particularly in patients without a prior cesarean, in whom these exposures may represent the primary source of uterine disruption.

### 1.3. Clinical Significance

PAS disorders can present life-threatening complications throughout the peripartum period, often requiring urgent intervention, including blood transfusions and hysterectomy. PAS is a leading cause of peripartum hysterectomy, significantly contributing to maternal morbidity and mortality. In a comprehensive meta-analysis, the pooled incidence for peripartum hysterectomy in PAS cases was 52.2% (95% CI 38.3–66.4), with 46.9% (95% CI 34–59.9) of cases requiring transfusion due to hemorrhage [8]. Hysterectomy is more frequently required in cases with greater placental invasion and is often the definitive treatment to control life-threatening bleeding. Massive hemorrhage commonly occurs during or immediately after delivery and can lead to severe complications, including disseminated intravascular coagulopathy (DIC), hemorrhagic shock, and multisystem organ failure [17]. The pooled estimate for maternal mortality in PAS cases is 0.5% (95% CI 0.06–0.69), with lower mortality rates observed in patients managed in referral centers with multidisciplinary teams and diagnosed prior to delivery [1].

Placenta percreta, the most severe form of PAS, is associated with significantly higher maternal morbidity compared to the less invasive subtypes. In a retrospective study of 156 patients with PAS, the composite morbidity rate was significantly higher in the percreta group compared to the accreta group (86.3% vs. 26.7%, *p* < 0.001) [2]. Placenta percreta involves invasion through the entire myometrium and may reach surrounding structures, such as the bladder, ureters, and bowel. This extensive invasion can cause local destruction and severe complications, including massive hemorrhage, DIC, adult respiratory distress syndrome, and kidney failure [18]. Surgical management of placenta percreta is particularly challenging and can result in injury to surrounding organs, particularly the bladder and ureters [13].

PAS is also associated with significant fetal complications, primarily due to the increased risk of preterm birth. Vinograd et al. found that PAS significantly increases the likelihood of preterm delivery before 34 and 37 weeks (OR 1.4; 95% CI 1.1–1.7) and perinatal mortality (OR 8.2 95%; CI 6.4–10.4) [14].

### 1.4. Pathophysiology

The pathophysiology of PAS disrupts the normal attachment of the placenta to the uterine wall, leading to maternal complications during placental separation after delivery. A definitive diagnosis of PAS is achieved through histological examination of the uterine wall, which classifies the extent of villous invasion into the myometrium. The prevailing hypothesis regarding the development of PAS is that of a defect in the decidua basalis, specifically at the endometrial-myometrial interface [15].

#### 1.4.1. Normal Placentation

The endometrial cycle consists of three phases: the proliferative phase, the secretory phase, and menstruation. In a normal 28-day cycle, days 5–14 make up the proliferative phase, where rising estrogen levels lead to proliferation of the glands and stromal matrix. Ovulation occurs on day 14, after which the corpus luteum is formed and is responsible for producing progesterone during the secretory phase (days 15–28). If fertilization does not occur, the corpus luteum degenerates, causing a fall in progesterone levels, which leads to vascular constriction and subsequent endometrial shedding [16]. After ovulation, during the mid-secretory phase, high levels of progesterone lead to the differentiation of endometrial stromal cells (ESC), secretory transformation of uterine glands, increased localization of uterine natural killer (NK) cells, and promotion of angiogenesis. This process is known as decidualization, a critical step in ensuring successful implantation of the blastocyst into the uterine epithelium [19].

During implantation, the blastocyst attaches to the uterine epithelium and triggers the differentiation of the outer layer known as the trophoectoderm. This layer differentiates via two pathways: villous and extravillous [20]. In the villous pathway, cytotrophoblasts (CTBs) fuse to form syncytiotrophoblasts (STBs), which are involved in the production of human chorionic gonadotropin (hCG) and interact directly with endometrial blood supply to facilitate the exchange of nutrients and waste [20,21,22]. In the extravillous pathway, CTBs can form anchoring villi, which directly adhere to the uterine wall. From the anchoring villi, two types of extravillous trophoblasts (EVTs) form: interstitial (iEVTs) and endovascular (enEVTs). iEVTs anchor the blastocyst into the maternal decidua. enEVTs penetrate the uterine spiral arteries, converting them into low-resistance, high-capacity utero-placental vessels, ensuring increased blood flow to meet the growing demands of the fetus [20,23].

The invasion of EVTs is a tightly regulated process, as insufficient invasion may lead to fetal growth restriction and excessive invasion may lead to placenta accreta spectrum [20,24]. Healthy decidualized stromal cells secrete two important proteins, Prolactin (PRL) and Insulin-Like Growth Factor Binding Protein-1 (IGFBP-1), which have been suggested to regulate trophoblast growth and invasion, modulate uterine NK cells protecting the embryo from maternal immune attack, and promote angiogenesis [25]. The depth of invasion is regulated by a balance between matrix metalloproteinases (MMPs), secreted by trophoblasts to degrade the decidual extracellular matrix, and tissue inhibitors of metalloproteinases (TIMPs), released by endometrial stromal cells (ESCs) to limit excessive invasion [26].

#### 1.4.2. Developmental Origins of Abnormal Placentation

Current understanding identifies a damaged uterine wall, commonly due to prior uterine surgery, as the primary initiating factor [27]. This injury disrupts normal decidualization, leading to the loss of regulatory signals, altered gene expression, inflammation, and the formation of a rigid, collagen- and fibrin-rich extracellular matrix [28]. Overall, this aberrant structural and biochemical environment results in unregulated trophoblast invasion beyond the decidua.

In PAS, the regulation of trophoblast invasion is disrupted as a result of defective or absent decidualization secondary to uterine scarring from prior cesarean sections, myomectomy, or curettage [29]. In primigravid women without prior uterine scarring, PAS is extremely rare [30]. As a result of uterine scarring, the key structural barriers between the placenta and uterus, namely the decidua basalis and Nitabuch’s layer, are often absent secondary to absent endometrial decidualization [31,32]. In a normal pregnancy, the decidua basalis forms the interface between the maternal uterus and placenta while enabling placental separation during delivery. Nitabuch’s layer provides a physical barrier that limits excessive trophoblast penetration into the myometrium [32]. Importantly, PAS does not result from hyperactive trophoblasts but rather from a defective decidual-myometrial interface over areas of uterine scarring, resulting in the loss of regulatory signals (i.e., TIMPs, Prolactin, IGFBP-1) that normally limit trophoblast invasion [26,28,33,34].

In the absence of the decidual layer, EVTs invade directly into the myometrium as a result of various factors. In early pregnancy, physiologic hypoxia normally directs trophoblast migration towards the spiral arteries within the endometrium, where they initiate maternal vascular remodeling as enEVTs. In regions lacking normal vasculature, such as uterine scars, this target zone is absent, leading to persistent hypoxic conditions that sustain the invasive EVT phenotype and promote deeper infiltration into the myometrium [35]. As a result, disorganized vascular remodeling in PAS creates hypervascular zones composed of fragile vessels that predispose to hemorrhage during delivery [36].

#### 1.4.3. Molecular and Cellular Mechanisms Involved in Trophoblast Invasion and Uterine Wall Remodeling

Beyond hypoxia-driven mechanisms, the scarred uterine environment itself contributes to persistent invasion through profound extracellular matrix remodeling. Uterine scarring results in an environment characterized by excessive collagen deposition, anisotropic collagen fibril architecture, increased mechanical stiffness, and significant fibrinoid matrix deposition [3,37].

In PAS, EVTs exhibit an abnormally sustained endothelial-to-mesenchymal transition (EMT), a process that is typically transient during early placentation. This transition shifts trophoblasts toward a mesenchymal phenotype, enabling their migration into the decidua and remodeling of the spiral arteries. In PAS, the EMT persists throughout pregnancy, indicating a failure to revert to a non-invasive state [38].

The mechanisms that trigger this upregulation and thus control EMT are not fully understood but involve multiple signaling pathways, including transforming growth factor-beta (TGF-β) and Wnt, both of which impact trophoblast differentiation and invasion [36]. It is suggested that altered TGF-β and Wnt signaling pathways in PAS enhance type I collagen production and promote myofibroblast activation, resulting in extracellular matrix remodeling and increased stiffness at the scar site [36]. Recent studies have also shown that scarred decidua exhibit increased expression and activity of Piezo1, a mechanosensitive ion channel in endometrial stromal fibroblasts (ESFs). Mechanical stress on the Piezo1 channels triggers the production of pro-inflammatory cytokines, IL-8 and G-CSF, which are key mediators in the recruitment of EVTs to the scar site [37]. Thus, dysregulation of these pathways promotes persistent EMT, further contributing to uncontrolled invasion.

Additionally, EVTs from PAS placentas show overexpression of the transcription factor ZEB, which drives mesenchymal differentiation, along with downregulation of E-cadherin (CDH1), a mediator of epithelial cell–cell adhesion [39]. Several additional biomolecules have been identified as involved in both PAS and metastasis, including apoptosis and senescence regulators, reinforcing the connection between PAS trophoblasts and their invasive, tumor-like behavior [40,41].

The ECM in normal decidua supports trophoblast migration while restraining excessive penetration. In normal placentation, matrix metalloproteinases (MMPs), particularly MMP-2 and MMP-9, degrade ECM components to enable EVT entry into maternal tissue. In PAS, these MMPs are upregulated, contributing to the degradation of the ECM and promoting EVT invasion [42]. Uterine scars contain excessive collagen deposition, fibrin accumulation, increased ECM rigidity, and impaired structural integrity, which contribute to anchoring of trophoblasts into the myometrium [28]. The genesis of this regulatory dysfunction both at the EVT and ECM has been increasingly attributed to a decreased inhibitory signal from the uterine wall, described as a “loss of boundary limits” in the decidua due to prior uterine scarring [28].

Angiogenesis and immune modulation are also dysregulated in PAS. The implantation site is hypervascular due to elevated pro-angiogenic factors (including VEGF and Angiopoietin-2) and reduced anti-angiogenic signals (including sFlt-1 and VEGFR-2) [27]. Incomplete spiral artery remodeling disrupts the normal transformation of maternal vessels by EVTs into low-resistance channels, resulting in high-velocity blood flow, oxidative stress, and disorganized, leaky vasculature [36].

**Figure 1:**
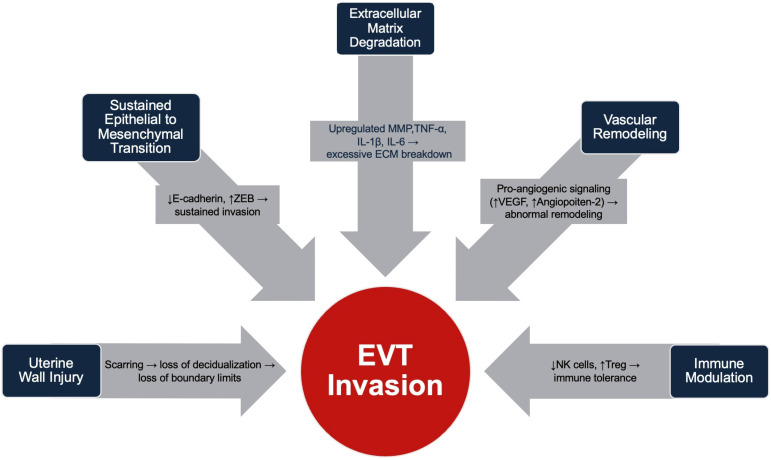
Summary of physiologic changes that promote EVT invasion in Placenta Accreta Spectrum.

Immune changes include reduced decidual NK cells and increased regulatory T cells and macrophages, creating a local region tolerant to invasive trophoblasts [27]. Elevated pro-inflammatory cytokines (TNF-α, IL-1β, IL-6) further promote trophoblast recruitment and ECM degradation [41]. Together, these alterations permit deep placental invasion and immune evasion in PAS. The changes that promote EVT invasion are outlined in Figure 1.

#### 1.4.4. Implications on Fetal Development

Beyond the pathophysiology of PAS development, it is also important to consider the implications of PAS on fetal development. In a prominent multicenter study in 2022 performed on a cohort of 865 subjects, Detlefs et al. examined the potential relationship between PAS severity and the weight of the neonate in the context of gestational age. An infant that was small for gestational age (SGA) was defined as less than the 10th percentile for weight. It was found that the relative risk of a neonate born SGA to a patient with placenta increta or percreta did not differ from accreta [43]. In another study of 171 patients with placenta previa and PAS and 146 patients with placenta previa but without PAS, fetal ultrasounds were performed at two timepoints: between 20 and 24 weeks gestation and 30–34 weeks. It was found that SGA rates did not differ between the PAS and non-PAS groups on the first or second ultrasound. This revealed that PAS was not associated with the delivery of SGA infants [44].

SGA occurs when a fetus experiences intrauterine growth restriction, meaning the fetus did not receive the necessary oxygen required for proper growth and development. Due to the absence of significant difference in delivery of SGA neonates to patients with PAS and without it, it has been surmised that PAS is not a cause for intrauterine growth restriction [45].

Valuable to note, a retrospective study performed at a single center between 2011 and 2022 evaluated adverse outcomes in neonates born to PAS patients, including umbilical artery pH of <7.0 and rates of hypoxic–ischemic encephalopathy and seizures. It was determined that in 265 patients with PAS compared with 1382 controls, the rate of composite adverse neonatal outcomes in PAS was significantly higher (33.6% vs. 18.7%, respectively, *p* < 0.001). A lower Apgar score of <7 at 5 min after birth and neonatal Intensive Care Unit admission were specifically found to be independently associated with PAS. Therefore, although infants of PAS groups may not experience intrauterine growth restrictions, PAS has been associated with greater rates of adverse outcomes for infants [46].

### 1.5. Diagnosis

While postnatal histological analysis remains the gold standard for confirming PAS, antenatal diagnosis, primarily through ultrasonography, has been strongly associated with better outcomes [47,48,49]. PAS is often asymptomatic and painless, but in severe cases such as placenta percreta, the placenta may invade adjacent organs, leading to symptoms like abdominal pain, hemoperitoneum, and hematuria [13,50,51]. Current guidelines recommend screening for PAS in all high-risk patients, particularly those with a history of cesarean delivery or placenta previa [52].

Ultrasound is the first-line imaging modality for the antenatal diagnosis of PAS. A meta-analysis of 3707 pregnancies reported that ultrasound had a sensitivity of 90.72% and a specificity of 96.94% for the diagnosis of PAS [53]. However, this high accuracy may be overestimated since the studies primarily involved patients with significant risk factors. In contrast, a prospective study of 174 low-risk pregnancies concluded that, without these risk factors, the independent predictive value of specific ultrasound findings is less reliable [54]. Furthermore, ultrasound’s effectiveness in diagnosing PAS has been shown to decrease in earlier stages of pregnancy, in cases with less severe invasion, and when the placenta is located posteriorly [55,56,57]. Although ultrasound can confirm the diagnosis of PAS, it cannot accurately predict the depth of invasion and thus the subtype [58]. However, antenatal diagnosis of PAS can help plan for cesarean delivery at the 34th or 35th week of gestation.

Classic ultrasound findings associated with PAS include placental lacunae, myometrial thinning, bladder-wall interruption, and subplacental hypervascularity [57]. One study found that 94% of patients with PAS had three or more of these classic signs, although the study was limited to patients with placenta previa, which may limit the generalizability of these findings [57].

The reliability of ultrasound diagnosis is influenced by multiple factors, including operator expertise, patient characteristics, and placental location. Image quality can vary depending on the approach and equipment used. FIGO guidelines recommend that examinations be initiated with a transabdominal scan, using a convex probe (3–5 MHz). Imaging resolution can be improved with the use of a higher frequency probe (5–9 MHz) or a linear transducer, particularly in cases of anterior placenta where near-field resolution is more important than depth of penetration [58]. In women with a history of low-lying previa placenta, it is recommended that the urinary bladder be adequately filled (200–300 mL) to allow optimal visualization of the lower uterine segment and the uterovesical interface during ultrasound screening for PAS. In patients with a high BMI, image quality can be limited with a transabdominal approach; this can be enhanced by lifting the pannus and scanning beneath it [59]. Operator-dependent factors include excessive probe pressure, which may result in loss of the retroplacental clear zone, one of the signs of invasive placentation. Assessment of vascularity in the lower uterine segment is highly subjective and depends on the appropriate power Doppler gain setting and correct color flow velocity scale, which is often difficult even for experienced sonographers. Inappropriate adjustment may result in either overestimation or underestimation of the vascularity [59].

To standardize US findings, Rac et al. developed the placenta accreta index (PAI), a standardized scoring system derived from a single-center retrospective cohort study of 184 women with ≥1 prior cesarean delivery and a third-trimester diagnosis of placenta previa or low-lying placenta. Five variables were identified as strong predictors of histologically confirmed PAS: number of prior cesarean deliveries, placental location, presence of lacunar spaces, smallest sagittal myometrial thickness, and presence of bridging vessels. Each parameter was weighted to generate a PAI score ranging from 0 to 9, estimating a probability of invasion from 2 to 96%. In receiver operating characteristics (ROC) analysis, the model achieved an area under the curve of 0.87 (95% CI 0.80–0.95). The final model is derived from a cohort limited to women with prior cesarean delivery and third-trimester placenta previa with complete imaging data available in only 88 of 184 cases, so its applicability to lower-risk populations and different placental locations remains uncertain [4].

Similarly to Rac et al., a prospective cohort of 100 high-risk pregnancies by Abu Hashim et al. reported an AUC of 0.84 for the Placenta Accreta Index, with an optimal cut-off index of 5.37 yielding 84% sensitivity and 76% specificity [60]. However, this study was also limited by a small high-risk cohort with a history of prior cesarean section and placenta previa. The high prevalence of PAS within the selected populations in these studies may overestimate the diagnostic accuracy and generalizability to lower-risk populations. 

#### The Role of MRI

MRI is increasingly utilized in the antenatal diagnosis of PAS, particularly when ultrasound results are inconclusive or when a more detailed assessment of placental invasion is required. MRI is especially useful in cases involving posterior placentas or when assessing the extent of abdominal invasion in more severe cases of PAS [61]. MRI for PAS is typically performed without gadolinium using multiplanar T2-weighted sequences between 28 and 32 weeks of gestation. MRI features that have been associated with PAS include uterine bulging, heterogeneous placenta, placental bands, and focal interruptions in the hypointense myometrial border [62]. A systematic review and meta-analysis found that MRI has similar sensitivity and specificity to ultrasound for the antenatal diagnosis of PAS [63]. While the evidence supporting its use is not conclusive, MRI is valuable in cases with inconclusive ultrasound findings or to better visualize placental extension, and, when used with ultrasound, is likely to optimize both diagnostic accuracy and surgical management of PAS [64].

## 2. Management and Considerations

### 2.1. Preoperative

The anesthesia team should be notified of patients with PAS in advance of the scheduled delivery date, typically planned for at the 34th week of gestation [65]. Careful planning should be based on the patient’s current medical status, history, and obstetric risk factors that increase the likelihood of hemorrhage during cesarean delivery. Women undergoing urgent delivery for adherent placenta are at increased risk for maternal morbidity compared to those undergoing planned deliveries at the standard 34th or 35th week of gestation [66]. Early anesthesia consultation and proper preparation prior to delivery can help reduce intraoperative complications. Emergency cesarean section can occur in 35% of patients with PAS, with 80% occurring prior to 34 weeks [61]. This highlights the importance of careful antenatal planning to reduce the risk of complications, in addition to ensuring appropriate medical resources are readily available for immediate unplanned deliveries. The ideal location for surgery is largely dependent on an individual center’s resources but should have access to the surgical tools required for a cesarean hysterectomy, access to rapid transfusion equipment, neonatal resuscitation equipment, and interventional radiology for possible uterine artery embolization or intra-arterial balloon occlusion [67]. Additionally, adequate preoperative coordination with a multidisciplinary team has been shown to decrease blood product requirements, intraoperative blood loss, and maternal morbidity [68,69].

#### 2.1.1. History and Physical Exam

Preoperatively, patients should be assessed for current medications, allergies, prior complications with anesthesia, and past medical history. Additionally, more detail should be focused on comorbidities that commonly arise during pregnancy. Women become more hypercoagulable during pregnancy and are 4 to 5 times more likely to experience a thromboembolic event during pregnancy compared to when they are not pregnant [59]. The most important factors to assess during this part of the exam include a history of thrombophilia, such as Factor V Leiden, Protein C/S deficiency, and antiphospholipid syndrome. Women with a high risk of VTE are recommended to be on LMWH during pregnancy.

A detailed obstetric history must be obtained by the anesthesia team, focusing on factors that may increase the risk of intraoperative complications. Risk factors for PAS include prior cesarean deliveries, advanced maternal age (≥35 years), multiparity, prior uterine surgery, and abortions. Pre-existing conditions such as preeclampsia, gestational thrombocytopenia, and HELLP syndrome all increased the risk of hemodynamic compromise. Ultrasonographic findings are valuable for determining the extent of a morbidly adherent placenta and can help guide preventative measures to manage the risk of postpartum hemorrhage (PPH) [60]. It is important to consider the patient’s surgical history, including abdominal and cesarean sections, as postoperative adhesions from prior surgeries can increase operating time [62]. Careful consideration of past surgical history should be made when determining the anesthesia plan.

A general physical exam should assess the airway, heart, lungs, and extremities. A thorough airway assessment includes assessing neck flexion, circumference, and Mallampati classification. These assessments can help the anesthesia provider guide their approach in the case in which a difficult airway may arise. It is important to thoroughly examine the spine for deformities that may interfere with neuraxial anesthesia. General labs can be ordered prior to the procedure. In addition to a focused physical exam, the American Society of Anesthesia (ASA) recommends a baseline complete blood count, blood type and screen, and perianesthetic records of fetal heart patterns be obtained prior to induction of anesthesia [63].

#### 2.1.2. Anesthesia Planning and Maternal Considerations

A mother with suspected PAS undergoing a planned cesarean section should understand the high risk of an accompanying hysterectomy. In the case where bleeding cannot be controlled, a total hysterectomy is recommended [64]. Only 53% of women are diagnosed with PAS before cesarean delivery, and 33% experience significant blood loss, with 92% ultimately requiring a hysterectomy [4]. Hysterectomy is irreversible, and the psychological effects can be long-lasting, particularly for premenopausal women with plans to have more than one child. Women diagnosed with PAS prior to cesarean delivery are at increased risk of developing post-traumatic stress disorder as a result of a traumatic delivery. Therefore, it is important to coordinate for psychological evaluation and support services prior to surgery [70,71]. It is important to inform the patient that, depending on their wishes, conservative measures can be considered. These may include leaving the placenta in situ and/or performing a focal resection if less than 50% of the adherent placenta is attached to the anterior wall of the uterus [72]. However, there are yet no clear criteria on when and how to implement conservative management, and many centers do not offer this option due to the complexity and risks involved [73]. Evidence has shown that conservative treatment does not appear to compromise fertility in women with PAS but can increase the risk of recurrent PAS in subsequent pregnancies [74].

With the increased risk of hemorrhage, patients must understand that a blood transfusion may be required. Patients should understand the possible risks of transfusions, such as febrile reactions, anaphylactic reactions, and bacterial infections. Special considerations should be made for patients who are Jehovah’s witnesses, and documentation of which products they are willing to receive should be prepared. In such cases, effort should be made to maximize preoperative hemoglobin levels with iron supplementation and erythropoietin [75].

The anesthesiology team should discuss the differences between neuraxial anesthesia and general anesthesia in patients with suspected PAS. It is essential that the mother understands the risks and benefits associated with each type of anesthesia and is directly involved in the decision-making process. Neuraxial anesthesia allows mothers to be awake during the birth and minimizes the fetuses’ exposure to anesthetic agents. However, if hemorrhage cannot adequately be controlled during the surgery, conversion to general anesthesia is recommended. General anesthesia provides a secure airway from the beginning of the operation and allows for controlled ventilation. One important risk for general anesthesia is that it can potentially decrease uterine tone, increasing the risk of hemorrhage in PAS patients [76]. Some mothers may be concerned about a 2016 FDA communication regarding potential risks of general anesthesia during pregnancy, particularly its effect on fetal brain development. To date, well-controlled human studies have not demonstrated a significant risk to the fetus during general anesthesia [77]. The final decision on the type of anesthesia should be guided by the anesthesiologist’s clinical judgment, taking into account the specifics of the patient’s condition, including airway risk and the risk for hemorrhage, and their wishes, as well as the preferences and policies of the medical facility. In decisions between neuraxial and general anesthesia, it is important to note that confounding and bias are inevitable. Different studies measure different outcomes, including neonatal outcomes such as Apgar scores, maternal outcomes such as ICU admission and length of stay, and complications including infections and transfusions, which can make direct comparisons challenging. Additionally, selection bias may affect the results of retrospective studies.

### 2.2. Intra-Operative

#### 2.2.1. General Anesthesia

General anesthesia is recommended in cases where there is predicted massive hemorrhage, such as suspected PAS, and in patients where hemodynamics may be difficult to manage [78,79,80]. In emergency scenarios where there may not be enough time to achieve a neuraxial block, general anesthesia may be selected [81]. General anesthesia offers various benefits, such as allowing for the early security of an airway, ventilation control, and increased general patient comfort. The range of patients undergoing primary general anesthesia for PAS is between 9% and 76% in the United States and varies greatly from center to center [67]. Some retrospective studies have indicated that overall general anesthesia use has declined over time in women undergoing cesarean section [81,82]. While considered safe, general anesthesia has been associated with a 1.7-fold increased risk of maternal morbidity in comparison to neuraxial anesthesia for women undergoing cesarean delivery, mainly attributed to airway compromise [83].

The anesthesiologist must be aware of several risks of general anesthesia in obstetric patients. General anesthesia is accompanied by difficult intubation, airway maintenance, and a high risk of pulmonary aspiration [84,85]. Mothers undergoing cesarean section under general anesthesia are seven times more likely to have a failed intubation in comparison to non-pregnant patients [83]. A retrospective study by Aziz et al. showed that video laryngoscopy was used in obstetric patients with difficult airways and allowed for a higher rate of successful intubation on the first attempt [86]. Several studies have reported increased hemorrhage-related mortality and increased transfusion requirements in patients undergoing cesarean sections with general anesthesia compared to those with regional anesthesia [76,87]. One study by Hong et al. was specific to PAS patients but reported no significant differences in reported blood loss between the two modes of anesthesia [76]. In a recent retrospective cohort study by Nguyen et al., patients with PAS undergoing cesarean hysterectomy showed no differences in blood loss or transfusion requirements between those receiving general anesthesia and those undergoing neuraxial anesthesia [77]. This study also reported more postoperative respiratory complications in the general anesthesia group. The inconsistencies between these studies may suggest a high level of selection bias. It has been suggested that increased blood loss could be a result of the halogenated inhaled anesthetics leading to uterine atony [88].

#### 2.2.2. Considerations for Hemorrhage

Hemorrhage is the leading cause of maternal death worldwide [89]. It is therefore important to manage hemorrhage with a combination of monitoring, resuscitation, and hemostasis. The initial assessment of a patient with obstetric hemorrhage includes emptying the patient’s bladder, ensuring that there are no genital tract lacerations, estimating blood loss, and assessing the hemodynamic status [80]. The goal of resuscitation is to maintain oxygenation and tissue perfusion by restoring circulating blood volume. Wide-bore IV access with a high flow of warmed crystalloid fluid should be infused until blood products are available to be transfused. In the anticipation that vasopressors may be needed, central venous access should be considered. Using a left lateral tilt for uterine displacement should be performed to allow preload to the right heart [90].

Extensive hemorrhage requiring blood transfusion occurs in 47% of patients with PAS [8]. This demonstrates the need for careful collaboration from the anesthesia team and the blood bank to ensure the availability of blood products. A type and screen and crossmatch should be attained prior to surgery since these patients are at higher risk of hemorrhage. There are currently no standard transfusion protocols for PAS, but a sample recommendation could include 6 units of packed red blood cells (PRBCs), 4 units of fresh frozen plasma, and 1 unit of platelets. Over 41% of patients undergoing hysterectomy for PAS experience estimated blood loss ≥5000 mL, with those diagnosed prenatally via ultrasound experiencing higher amounts of bleeding [91]. A meta-analysis of RBC transfusion in PAS patients found that the mean number of units transfused was 6.61 units (95% CI 4.73–8.48; *n* = 220 patients). Therefore, having 6 units of RBC available for potential transfusion may be recommended [92]. In order to prevent unnecessary blood transfusion intraoperatively, screening for anemia in the antenatal period to optimize the patient’s hemoglobin preoperatively should also be performed [80].

#### 2.2.3. Conservative and Expectant Management 

While hysterectomy remains the standard management for PAS, several uterine-preserving strategies have been described in the literature [93,94,95]. These include both surgical approaches and adjunctive use of interventional radiology for uterine devascularization or embolization. Uterine-preserving strategies generally consist of focal resection of the adherent placental tissue while retaining the uterus or expectant management with the placenta left in situ. Recent evidence suggests that conservative, uterus-preserving strategies may reduce operative morbidity for selected PAS cases when managed in experienced, multidisciplinary centers. A meta-analysis by Hessami et al. (16 studies, *n* = 2300) found that, compared with cesarean hysterectomy, management with the placenta left in situ or via local resection was associated with lower estimated blood loss, with mean differences of 973.5 mL and 739.7 mL, respectively. Additionally, they also found that, compared to hysterectomy, focal resection was associated with decreased intraoperative pRBC requirement (mean difference −1.54 units, 95% CI 1.06–2.01). The main limitations of this meta-analysis include its reliance on retrospective observational studies, absence of consistent histologic confirmation of PAS, and inability to distinguish between planned and emergent cesarean hysterectomies following conservative management [96].

The International Society for Abnormally Invasive Placenta recommends that focal resection be considered when the adherent area is less than 50% of the anterior wall of the uterus [83]. In women wishing to maintain fertility or where extensive invasion is anticipated to result in hemorrhage, leaving the placenta in situ may be considered as an alternative approach [83]. Retention of the placenta is not without risk; delayed hemorrhage, disseminated intravascular coagulation, and sepsis have been reported as major complications [97,98]. In a multicenter study of 167 women in France, the rate of hysterectomy in women who underwent expectant management was 44%. Delayed hysterectomy was observed in a subset of patients (*n* = 18), at a median interval of 22 weeks, with the leading cause being secondary postpartum hemorrhage (44.4%) [97]. While this study is limited to a small sample, similar rates were observed in a systematic review by Clausen et al., where 58% of women with a retained placenta required delayed hysterectomy, in some cases as late as nine months postpartum [20]. Another option for patients undergoing expectant management includes delayed interval hysterectomy, especially in patients who may be at risk for hemorrhage if hysterectomy is performed at the time of cesarean section. A retrospective study by Zuckerwise et al. comparing delayed vs. immediate hysterectomy in patients with antenatal diagnosis of PAS found that patients that underwent delayed hysterectomy experienced lower blood loss and required fewer blood products [99]. The conservative methods described are mainly described in retrospective studies, and their use should be reserved for carefully selected patients who may desire fertility or where the risk of hemorrhage is high.

#### 2.2.4. Interventional Radiology

For IR-capable centers, balloon occlusion catheters or uterine artery embolization (UAE) may be performed to reduce uterine blood flow and blood loss prophylactically during hysterectomy or used emergently if significant bleeding occurs intra- or postoperatively. Balloon occlusion catheters may be placed at the level of the internal iliac, common iliac, or infrarenal aorta. Placement in the internal iliac requires bilateral femoral access for bilateral balloon placement and precise sizing, and various studies have shown little to no reduction in EBL compared to no balloon [100,101]. This limited efficacy is largely due to the extensive pelvic collateral circulation, making this approach unreliable for hemorrhage control. In contrast, the aortic approach via the common iliac arteries and infrarenal abdominal aorta has been associated with reduced blood loss and reduced hysterectomy rates [102,103,104]. The aortic approach allows for single femoral access, is faster to deploy, has shorter fluoroscopy time, and has decreased fetal radiation exposure compared to internal iliac [105]. Traditionally balloons are placed preoperatively and are inflated after delivery of the baby when hemorrhage risk is highest. Once hemostasis is achieved, the IR team can deflate the balloon and remain on standby if further bleeding occurs. Occlusion time is a critical consideration, as increased occlusion times can lead to irreversible tissue damage, and current trauma guidelines do not recommend exceeding 60 min in the distal abdominal aorta [106]. While there are no specific guidelines on internal iliac occlusion times, a mean of 19–33 min has been reported in one study, but it did not analyze a direct association between occlusion time and complications [107].

While occlusion balloons offer brief arterial blockage with immediate reperfusion once deflated, UAE delivers a more lasting hemostatic effect. The absorbable gelatin sponge that is used in the UAE provides temporary but sustained hemostasis that can reduce the risk of delayed postoperative bleeding, unlike the immediate reperfusion that follows balloon deflation. For these reasons, embolization may be preferred in PAS cases involving planned delayed hysterectomy or uterine preservation, or when coagulopathy increases the risk of recurrent hemorrhage. A combined approach using both balloon occlusion and embolization has also been utilized.

The most common complications associated with embolization are ischemic. Fortunately, because the pelvis is highly vascularized, it is generally resistant to ischemic injury after embolization. However, when embolization is performed with the intent of preserving the uterus, it is important to recognize that uterine ischemia or infarction with necrosis can occur. Even though the gelatin sponge material is designed to be temporary and only partially occlusive, excessive injection can override these properties. Management of uterine ischemia typically consists of supportive measures and targeted antibiotics according to institutional guidelines. Infarction is rare, at <1%, but may present with rapid clinical deterioration that may require emergent hysterectomy. Factors associated with uterine necrosis after UAE include limited collateral circulation and underlying sepsis. In general, any procedure that causes tissue ischemia increases the risk of infection, such as endometritis, especially in the setting of an open incision. For this reason, peri-procedural prophylactic antibiotics are recommended [108].

### 2.3. Postoperative

Due to the extensive surgery, the early postoperative period requires intensive hemodynamic monitoring for PAS patients. The monitoring may be provided in the intensive care unit (ICU) setting to ensure stabilization. According to the American College of Obstetricians and Gynecologists (ACOG), patients with known PAS are at risk for experiencing postoperative abdominopelvic bleeding, fluid overload from resuscitation, and other complications secondary to major blood loss.

**Figure 2:**
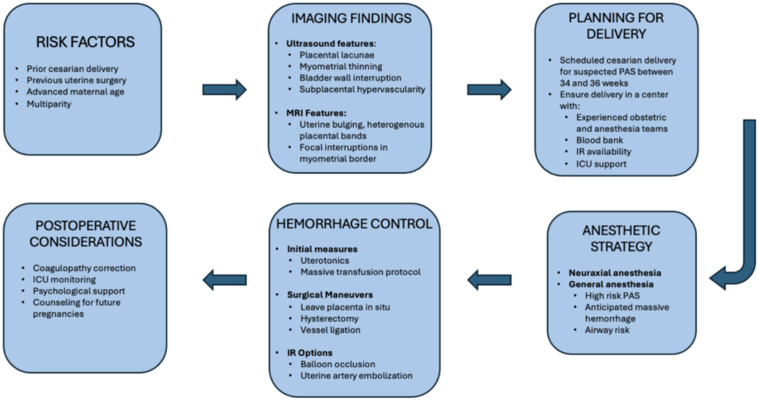
Management algorithm for Placenta Accreta Spectrum.

One observational study over a 15-year period examined peripartum maternal admission to the ICU. Among the leading causes of ICU admission, PPH led to the admission of 92 women (25.2%), of which 25 (27.2%) of cases were PAS-related complications. The correlation value of placenta percreta/increta was 0.96, meaning there was a strong increase in the number of PAS patients admitted to the ICU as the 15-year period progressed. PAS patient ICU admission represented the steepest trend of the other admission indications [109]. The need for critical care for the PAS patient population is evidenced by the rising incidence of PAS. The risk of hemorrhagic shock and instability requiring further RBC transfusion, and potentially continued mechanical ventilation, highlights the importance of continued monitoring and ICU-level care [110]. To support point-of-care decision-making, Figure 2 presents an integrated flowchart spanning suspicion for PAS, diagnostic imaging, delivery planning, anesthetic approach, and hemorrhage control strategies.

## 3. Unexpected Accreta Discovered Intra-Operatively

The incidence of unexpected PAS during delivery, though small, is not negligible and is associated with considerable morbidity. In a national retrospective cohort study of nearly 8.7 million vaginal deliveries, unexpected PAS was reported in 1 in 3797 deliveries [7]. Uterine anomalies were strongly associated with unexpected PAS (OR 6.23; 95% CI 4.20–9.26). Other factors, including older patient age, uterine myomas, early-term delivery, previous recurrent pregnancy losses, in utero growth restriction, and fetal demise, were also linked to unexpected PAS [7]. Patients with unsuspected PAS were significantly more likely to experience complications such as hemorrhage (65.2% vs. 4.1%), the need for blood transfusion (21.3% vs. 0.6%), hysterectomy (14.9% vs. <0.1%), coagulopathy (2.9% vs. 0.1%), and shock (2.9% vs. <0.1%) compared with those without PAS [7]. Additionally, a single-center study found that unexpected PAS cases had higher estimated blood loss (2.4 L vs. 1.7 L, *p* = 0.04) and required more red blood cell transfusions (4 vs. 2, *p* = 0.03) compared to expected PAS cases [111].

## 4. Future Fertility

Cesarean hysterectomy has been understood to decrease maternal morbidity and mortality following their delivery in the setting of PAS. Uterine-sparing techniques as conservative management are now used to conserve fertility and reduce surgical implications. However, the high risk of adverse outcomes may still preclude future fertility.

One meta-analysis involving 1458 participants established that the rate of PAS recurrence in subsequent pregnancies was 11.8% (95% confidence interval, 1.1–60.3; I2 = 86.4%). 1 in 4 subsequent pregnancies following conservative management via placenta left in situ were found to have considerable adverse maternal outcomes [43]. A retrospective study performed in Jerusalem studied 134 women who received conservative treatment for PAS between 1990 and 2000. PAS was found to have recurred in 62 (22.8%) of 272 subsequent deliveries as compared to 5 women (8.6%) of 266 women in the control group without a previous diagnosis of PAS. Early PPH occurred in 23 (8.6%) of deliveries with previous PAS, as compared to 7 (2.6%) in the control group. Recurrence and PPH are shown to be higher in women with previously diagnosed PAS, although most subsequent pregnancies for these women were successful [112].

## 5. Conclusions

PAS disorders remain a significant challenge in obstetric care due to their association with maternal morbidity and mortality, particularly due to massive hemorrhage and the need for peripartum hysterectomy. As the incidence of PAS rises, largely driven by increasing cesarean delivery rates, early recognition and multidisciplinary management are critical in reducing adverse outcomes. This review uniquely integrates the developmental biology and molecular mechanisms underlying PAS—including decidual defects, persistent EMT in trophoblasts, and scar-induced ECM remodeling—with detailed discussion of clinical diagnosis, perioperative planning, and hemorrhage management strategies. By linking these mechanistic insights to practical approaches in anesthesia, surgical planning, and interventional radiology, it provides a translational perspective that connects cellular pathophysiology to clinical care.

In summary, PAS reflects a convergence of molecular, surgical, and clinical factors. At the biological level, defective decidualization and aberrant trophoblast invasion, driven by sustained endothelial-to-mesenchymal transition, dysregulated TGF-β/Wnt signaling, and extensive ECM remodeling, promote the disorder’s pathogenesis. Clinically, prior cesarean delivery or other uterine surgery disrupts the decidual–myometrial interface, generating hypervascular, rigid, and highly permissive zones for invasive placentation. The combined profile of placenta previa and prior cesarean delivery remains the strongest predictor of PAS. Accurate diagnosis relies on imaging with ultrasound and MRI to characterize invasion depth, vascular patterns, and myometrial integrity, all of which inform surgical and anesthetic planning. Adjunctive interventional radiology strategies such as balloon occlusion and uterine artery embolization may further mitigate hemorrhage and reduce maternal morbidity. Ultimately, optimizing outcomes requires early identification and coordinated multidisciplinary management involving obstetrics, anesthesia, interventional radiology, and critical care, with individualized pathways ranging from fertility-preserving options to planned cesarean hysterectomy. Future research into extracellular matrix remodeling and immune-vascular interactions will be critical to developing predictive markers and novel therapeutic interventions aimed at reducing morbidity and mortality. Prospective cohort studies could track molecular, imaging, and clinical features from early pregnancy through delivery, while multicenter registries would provide the statistical power to validate risk models and evaluate outcomes of different management strategies. In the meantime, ensuring adequate antenatal planning, appropriate anesthetic strategies, and postoperative care is essential to improving the prognosis for patients affected by PAS.

## Figures and Tables

**Table 1:** Proposed subcategories of Placenta Accreta Spectrum based on histologic findings.

Proposed PAS Grade	Invasion Depth	Histologic Findings
**PAS Grade 1**	Noninvasive	Grossly adherent placenta by manual palpation. Myometrial cross sections show a smooth placental-myometrial interface and uniform myometrial thickness without thinning.
**PAS Grade 2**	Superficial invasion	Cross sections show an irregular placental–myometrial interface without involvement of the outer myometrium (i.e., preservation of >25% of the wall thickness relative to the uninvolved myometrium).
**PAS Grade 3A**	Deep invasion	Cross sections show an irregular placental–myometrial interface with involvement of the outer myometrium (i.e., with preservation of <25% of the wall thickness relative to the uninvolved myometrium). The serosa is intact.
**PAS Grade 3D**	Deep invasion with disruption of serosa	Deeply invasive placenta with disruption of the uterine serosal surface (D = deep invasion).
**PAS Grade 3E**	Deep invasion with adherent extrauterine structures	Placental invasion into adjacent organs or extrauterine fibroadipose tissue, confirmed by microscopy (E = extrauterine invasion).

## Data Availability

No datasets were generated or analyzed in this current review.
